# Thenar Muscle Atrophy: Clinical, Electrodiagnostic, and Ultrasound Features in 197 Patients

**DOI:** 10.3390/neurolint17120201

**Published:** 2025-12-11

**Authors:** Lisa B. E. Shields, Vasudeva G. Iyer, Stephen Furmanek, Yi Ping Zhang, Christopher B. Shields

**Affiliations:** 1 Norton Neuroscience Institute, Norton Healthcare, Louisville, KY 40202, USA; lbes@earthlink.net (L.B.E.S.); yipingzhang50@gmail.com (Y.P.Z.); 2 Neurodiagnostic Center of Louisville, Louisville, KY 40245, USA; pavaiyer@gmail.com; 3 Norton Data Science Core, Norton Healthcare, Louisville, KY 40202, USA; stephen.furmanek@nortonhealthcare.org

**Keywords:** neurology, thenar muscle atrophy, carpal tunnel syndrome, median nerve, electrodiagnostic study, ultrasound

## Abstract

Background/Objectives: Atrophy of the thenar muscles (abductor pollicis brevis [APB], opponens pollicis [OP], and flexor pollicis brevis [FPB]) is most commonly caused by carpal tunnel syndrome (CTS). It may also occur following injury to the recurrent motor branch of the median nerve, proximal median nerve neuropathy, medial cord/lower trunk plexopathy, T1 radiculopathy, ventral horn cell disorder at C8 or T1, disuse atrophy, or congenital aplasia. Clinical observation of flattening of the thenar eminence coupled with electrodiagnostic (EDX) and ultrasound (US) studies is valuable in determining the etiology of thenar atrophy. This study describes clinical, EDX, and US findings in a large cohort of patients with thenar muscle atrophy. Methods: This is a review of 197 patients (226 hands) with thenar atrophy who underwent EDX and US studies. Patients were divided into those with total thenar atrophy (all three thenar muscles were atrophic) or partial thenar atrophy (atrophy of one or two thenar muscles) based on clinical and US findings. Results: Of the 226 hands, 174 (77.0%) had partial thenar atrophy, 217 (96.0%) had sensory loss, and all hands demonstrated weakness of the APB and OP muscles on examination. A total of 220 (97.3%) hands had EDX evidence of severe median nerve entrapment at the carpal tunnel. The compound muscle action potentials (CMAPs) of the APB muscle and sensory nerve action potentials (SNAPs) were absent in 186 (82.3%) and 212 (93.8%) hands, respectively. US study showed hyperechoic APB and OP muscles in 225 (99.6%) hands. The Heckmatt grade, determined by US, was 3 in 152 (67.3%) hands, showing increased muscle echogenicity with loss of architecture and reduced bone reflection. Conclusions: In patients with thenar muscle atrophy, EDX studies were not always conclusive for confirming CTS due to an absence of SNAP and CMAP over the APB and second lumbrical muscles. In these cases, US is important to confirm the cause of thenar atrophy.

## 1. Introduction

**Figure 1:**
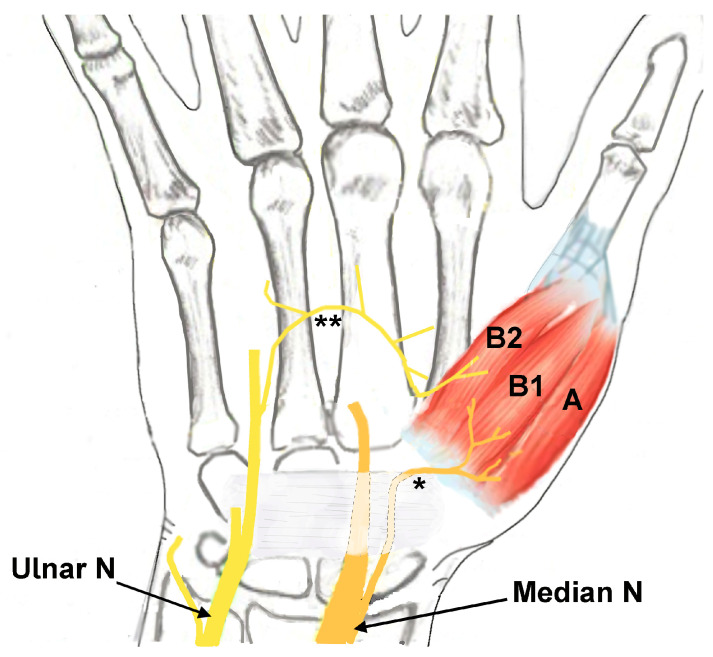
Drawing depicting the innervation of the thenar muscles (A: abductor pollicis brevis innervated by the median nerve; B1: flexor pollicis brevis (FPB) muscle superficial head innervated by the median nerve; B2: FPB muscle deep head innervated by the ulnar nerve). * Recurrent motor branch of the median nerve. ** Motor branch from the ulnar nerve to the deep head of the FPB muscle. Ulnar N: ulnar nerve. Median N: median nerve.

The abductor pollicis brevis (APB), opponens pollicis (OP), and flexor pollicis brevis (FPB) muscles comprise the thenar muscles and are located at the radial aspect of the palm [1,2]. The thenar muscles originate from the transverse carpal ligament and carpal bones and insert on the proximal phalanx of base of the thumb, except for the OP muscle, which inserts on the first metacarpal. The two heads of the FPB muscle have different innervations, with the deep head innervated by the deep branch of the ulnar nerve and the superficial head by the recurrent branch of the median nerve (Figure 1) [1,3,4]. The APB and OP muscles are innervated only by the recurrent branch of the median nerve (Figure 1). The primary role of the thenar muscles is operating the carpometacarpal (CMC) joint of the thumb, including precision pinching and grip strength [2,4,5].

Thenar muscle atrophy refers to flattening of the thenar eminence and may be classified as total (all three thenar muscles are atrophic) or partial (atrophy limited to one or two thenar muscles) (Table 1). In 1909, Hunt associated partial thenar atrophy with repeated occupational trauma against the motor branches of the median nerve distal to the ligamentum carpi transversum [6]. In 1939, Wartenberg reported seven new cases with slow development of partial thenar atrophy of the radial portion of the muscles of the thenar eminence involving only the APB and OP muscles. Four of these patients had bilateral atrophy [7]. Patients were referred for numerous conditions, including multiple sclerosis, syringomyelia, occupation neuritis of the median nerve, and neuritis of the brachial plexus.

While thenar muscle atrophy is most frequently caused by carpal tunnel syndrome (CTS), it may also occur following injury to the recurrent motor branch of the median nerve, proximal median nerve neuropathy, medial cord/lower trunk plexopathy, cervical radiculopathy of the C8 or T1 nerve roots, ventral horn cell disorder of C8 and T1, disuse atrophy, or congenital aplasia [5,8]. Other causes of thenar muscle atrophy include inflammatory or autoimmune diseases of the peripheral and central nervous system, such as Guillain–Barré syndrome and acute transverse myelitis, which can affect both the peripheral motor neuron and the central fibers [9]. Preferential atrophy of thenar lateral hand muscles has also been described in otherwise healthy aging subjects where the ‘senile hand’ or ‘split hand’ pattern was identified. These differential diagnoses highlight the need to consider age-related changes when interpreting thenar muscle thinning or electromyographic/findings [10]. Needle EMG is crucial in making the distinction between these varied conditions.

The incidence of thenar atrophy in patients with CTS ranges between 5 and 43%, with a higher incidence (62–100%) in severe CTS [11]. Thenar muscle atrophy has a specificity of 90–99% for the diagnosis of CTS, although sensitivity is only 12.6% [5]. Thenar muscle atrophy is most frequently seen in patients who have had signs and symptoms of CTS for a long duration [11]. Electrodiagnostic (EDX) studies consisting of nerve conduction velocity and needle electromyography are often used to detect abnormalities in the median nerve, such as axon loss, that may have led to thenar muscle atrophy.

Ultrasound (US) is a simple, rapid, readily available, accurate, cost-effective, and noninvasive method of visualizing the median nerve and its proximate structures [4,12]. US has the additional benefit of dynamic and functional muscle evaluation and assessment of superficial soft tissues [4]. Several studies have reported its use for detecting muscle changes in CTS [13] and for assessing thenar muscles [14,15,16,17]. In addition to evaluating patients with thenar atrophy, US is also important for confirming or ruling out CTS in patients with co-existing congenital thenar hypoplasia (Cavanagh syndrome) [18] or painless, nonhealing ulceration of the median nerve-innervated fingertips [19]. Magnetic resonance imaging (MRI) is valuable in detecting and characterizing hand muscle morphology, especially when thenar muscle atrophy is the main feature [20].

In this report, we evaluate clinical, EDX, and US findings in patients with thenar muscle atrophy. We discuss the mechanisms involved with total and partial thenar atrophy. The importance of US in cases when compound muscle action potentials (CMAPs) and sensory nerve action potentials (SNAPs) are absent is also highlighted.

## 2. Materials and Methods

Under an Institutional Review Board (IRB)-approved protocol, we performed a 5½ year (1 January 2020–30 June 2025) retrospective analysis of patients who were referred to our neurodiagnostic center for EDX studies in whom clinical examination showed thenar muscle atrophy. Our American Association of Neuromuscular and Electrodiagnostic Medicine (AANEM)-accredited neurodiagnostic center assesses approximately 1000 patients annually referred for EDX studies predominantly by hand surgeons and neurosurgeons. The EDX protocol includes nerve conduction and needle electromyography (EMG) studies. The US protocol consists of studying long-axis and short-axis images of the median nerve at the wrist and the forearm as well as the thenar muscles using an 8–18 or 6–15 MHz probe [21]. We included all patients with thenar muscle atrophy who underwent a clinical examination and EDX and US studies. The same electromyographer, a board-certified neurologist in electrodiagnostic medicine and clinical neurophysiology, performed the evaluations of all patients, which negated any inter-reliability bias.

### 2.1. Inclusion and Exclusion Criteria

Inclusion criteria were patients referred for EDX studies who exhibited atrophy of the thenar muscle. These patients required nerve conduction and needle EMG studies and US of the thenar muscles and median nerve. Exclusion criteria included patients who showed clinical and EMG patterns of significant involvement of other intrinsic hand muscles or proximal median nerve-innervated muscles.

### 2.2. Metrics Collected

The clinical metrics collected included (1) demographics, laterality (left, right, or bilateral), and hand dominance (right, left, or ambidextrous); (2) history of prior carpal tunnel release (CTR), diabetes mellitus, and pain or swelling of the thumb joints; and (3) clinical findings, including thenar muscle atrophy pattern (total, partial, or hypertrophic FPB muscle), atrophy of other intrinsic hand muscles, muscle strength, sensory loss, and other features such as hypoplastic thumb/thumb deformity, “split hand sign”, and Gilliatt Sumner hand”.

EDX studies were performed utilizing the standard protocol in our lab [22]. The EDX grading system used in our lab is simpler than the elaborate ones described by Bland et al. and Hirani et al. [23,24]. The median nerve motor conduction included estimation of the distal motor latency and conduction velocity across the forearm and amplitude of the CMAP. If no CMAP was recordable over the APB muscle, the study was repeated by placing the recording electrode over the second lumbrical muscle. If the FPB muscle was prominent, the recording electrode was placed over the FPB muscle and the CMAP was recorded by median and ulnar nerve stimulation at the wrist to determine the innervation pattern. The median nerve sensory conduction consisted of measuring the peak latency and amplitude of the SNAP over the 2nd and 3rd fingers after antidromic stimulation at the wrist. Needle EMG of the APB muscle determined the extent of motor unit loss and documentation of denervation changes.

The US evaluation consisted of the cross-sectional area (CSA) of the median nerve at the wrist (distal wrist crease) and at the mid-forearm. The thenar muscle was evaluated to document changes such as echogenicity in the APB, OP, and FPB muscles and to determine the Heckmatt grade [25,26].

Several scales were used to assess hand function in cases of thenar atrophy, to determine the presence of CTS, and to evaluate US findings. The MRC (Medical Research Council) scale was used to assess the muscle strength of the APB muscle. Clinical assessment of the severity of CTS was based upon the extent of muscle weakness and atrophy as well as sensory loss. An EDX scale was used to grade the CTS as mild, moderate, moderately severe, or severe based on the extent of involvement of the sensory and/or motor fascicles [21]. Thenar atrophy determined by US was classified as either total (APB, OP, and FPB muscles were hyperechoic with no normal muscle in the thenar area) or partial (APB and OP muscles were hyperechoic with partial atrophy of the FPB muscle). The cross-sectional area (CSA) of the median nerve was measured by the trace technique at the carpal tunnel (CT) inlet, exit, and at the mid-forearm [21,27].

### 2.3. Statistical Analysis

To compare the correlation between severity of CTS and Heckmatt score, the Spearman rank order correlation was calculated using an ordinal scale of severity (normal, mild, moderately severe, and severe). Two-sided tests of significance were performed with the null hypothesis that the Spearman correlation was equal to 0 [28]. *p*-values less than 0.05 were considered statistically significant.

### 2.4. Institutional Review Board Approval of Research

Informed consent was obtained from all patients. The WCG IRB determined that our study was exempt under 45 CFR 46.104(d)(4). The IRB number is 20250162, and the Ethic Approval Code is 01172025. IRB approval date was 17 January 2025.

## 3. Results

### 3.1. Demographics

A total of 197 patients had evidence of thenar muscle atrophy on clinical examination (Table 2). The mean age was 68.8 years (range: 15–92 years), and 100 (50.8%) patients were female. Thenar muscle atrophy was more common on the right side (109 [55.3%]), followed by the left side (59 [30.0%]), and there were 29 bilateral cases (14.7%). A total of 172 (87.3%) patients were right-handed. The side of thenar muscle atrophy corresponded to the dominant hand in 135 (68.5%) patients. Thirty-three (16.8%) patients had diabetes mellitus. Fourteen (6.2%) had previously undergone a CTR on the side of the thenar atrophy.

### 3.2. Neurological Examination

**Figure 2:**
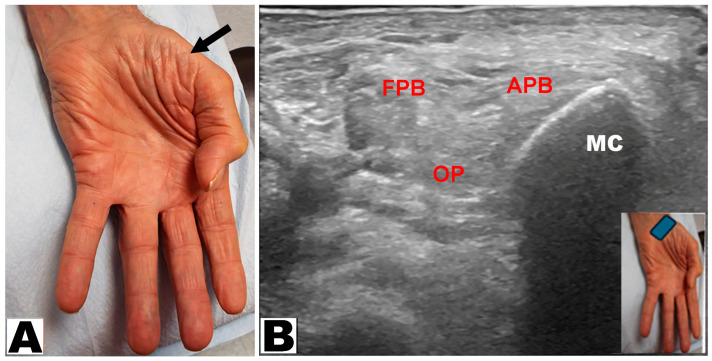
(**A**) Total atrophy of all thenar muscles of the hand (oblique arrow). (**B**) Total atrophy of the thenar muscles by ultrasound. APB: abductor pollicis brevis muscle. OP: opponens pollicis muscle. FPB: flexor pollicis brevis muscle. MC: metacarpal.

**Figure 3:**
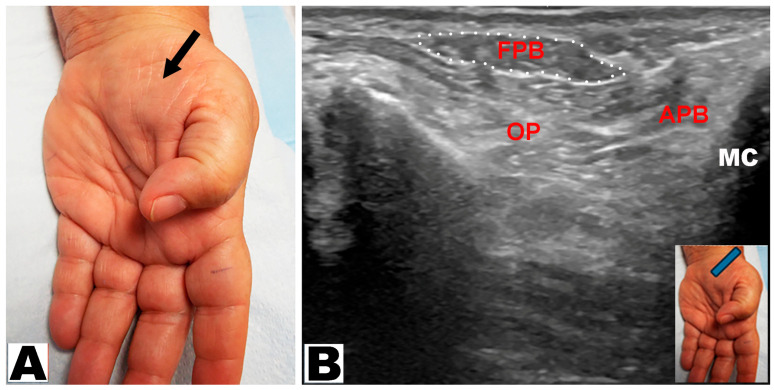
(**A**) Partial thenar atrophy: the flexor pollicis brevis muscle is visible (arrow) with atrophy of the abductor pollicis brevis muscle. (**B**) Ultrasound depicts partial thenar atrophy. The intact flexor pollicis brevis (FPB) muscle is encircled. APB: abductor pollicis brevis muscle. OP: opponens pollicis muscle. MC: metacarpal.

**Figure 4:**
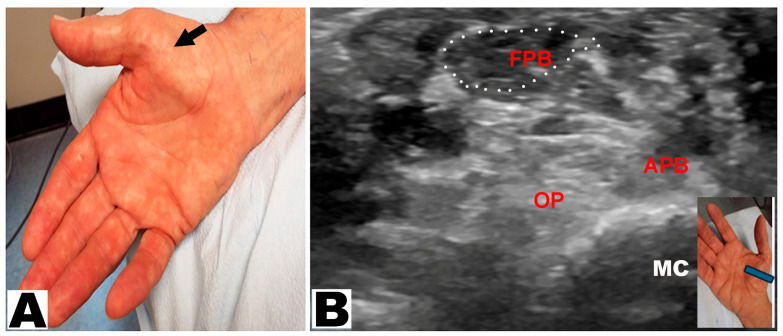
(**A**) Partial thenar atrophy with hypertrophy of the flexor pollicis brevis (FPB) muscle (oblique arrow). (**B**) Ultrasound study confirms partial thenar atrophy with a hypertrophic FPB muscle (encircled) and severe atrophy of the APB and OP muscles. The blue insert denotes the position of the ultrasound probe. APB: abductor pollicis brevis muscle. OP: opponens pollicis muscle. MC: metacarpal.

Thenar muscle atrophy was observed in a total of 226 hands (197 patients unilateral and 29 patients bilateral) (Table 3). Of the 226 hands, thenar muscle atrophy was partial in 174 (77.0%) hands and total in 52 (23.0%) hands (Figure 2A,B). The APB and OP muscles were weak in all (100%) hands. A prominent FPB muscle was noted in 44 (19.5%) hands, all of which had partial thenar muscle atrophy (Figure 3A,B and Figure 4A,B). A total of 217 (96.0%) hands had sensory loss.

### 3.3. Electrodiagnostic Studies

Most (220 [97.3%]) hands with thenar muscle atrophy had severe median nerve entrapment at the carpal tunnel according to EDX findings (Table 4). Mild and moderately severe entrapment at the carpal tunnel was only observed in three hands (1.3%) and one hand (0.44%), respectively. Thenar atrophy was not caused by CTS in two patients. One patient had evidence of disuse atrophy associated with rheumatoid arthritis, with evidence of multiple joint deformities. The other patient developed thenar muscle atrophy due to a cervical radiculopathy of the C8 and T1 nerve roots.

**Figure 5:**
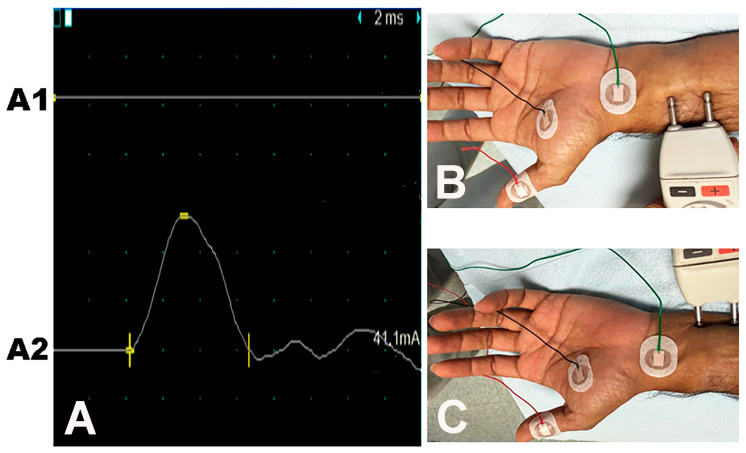
Median-ulnar flexor pollicis brevis (FPB) muscle study: the recording electrode is placed over the FPB muscle. Traces (**A**): (**A1**) No CMAP on median nerve stimulation. (**A2**) Normal CMAP on ulnar nerve stimulation. (**B**) Site of stimulation of the median nerve. (**C**) Site of stimulation of the ulnar nerve.

The CMAP of the APB muscle was absent in 186 (82.3%) hands (Table 5). Of the 188 hands that underwent EDX studies of the second lumbrical muscle, the CMAP was absent in 106 (56.4%) hands. Motor unit recruitment of the APB muscle was either absent (119 [52.9%] hands) or decreased (104 [46.2%]). Fibrillations/positive waves were observed in the APB muscle in 172 (76.4%) hands. An ulnar nerve neuropathy was detected by EDX studies on the side of the thenar muscle atrophy in 94 (41.6%) hands. Of these 94 hands, 59 (62.8%) had partial thenar muscle atrophy, while 35 (37.2%) were noted with total thenar muscle atrophy. Of the 59 hands with a combined partial thenar atrophy and ulnar neuropathy, 15 (16.0%) had hypertrophy of the FPB muscle. The SNAPs of the median nerve were absent in 212 (93.8%) hands (Table 6). Figure 5A–C depict a median and ulnar nerve study of the FPB muscle, showing absent CMAP on median nerve stimulation at the wrist and intact CMAP on ulnar nerve stimulation at the wrist.

### 3.4. Ultrasound Studies

**Figure 6:**
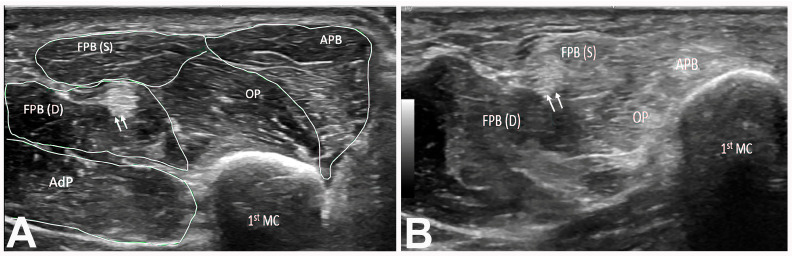
(**A**) Short axis of right thenar muscles (Heckmatt grade 1). The double arrow indicates the FPL tendon. (**B**) Short axis view of right thenar muscles (Heckmatt grade 3) showing hyperechoic APB, OP, and FPB(S) muscles. Note the normal appearance of the FPB(D) muscle. The double arrow demarcates the FPL tendon. 1st MC: first metacarpal bone. APB: abductor pollicis brevis muscle. OP: opponens pollicis muscle. FPB: flexor pollicis brevis (S: superficial head; D: deep head). AdP: adductor pollicis muscle. FPL: flexor pollicis longus.

The APB and OP muscles were hyperechoic in 225 (99.6%) hands, while the FPB muscle was hyperechoic in 29 (12.8%) hands (Table 7). Of the 29 hands with a hyperechoic FPB muscle, 24 (82.8%) hands had total thenar muscle atrophy and 5 (17.2%) had partial thenar muscle atrophy. A total of 104 (46.0%) hands had a wrist CSA between 10 and 20 mm^2^, and 119 (52.7) hands had a wrist CSA greater than 20 mm^2^. The forearm CSA was between 3 and 6 mm^2^ in 102 (45.1%) hands, between 7 and 10 mm^2^ in 104 (46.0%) hands, and greater than 10 mm^2^ in 20 (8.9%) hands. The Heckmatt grade determined by US was 3 in the majority (152 [67.3%)] of hands, with 204 (90.3%) hands having a grade of 3 or 4 (Figure 6; Table 8). Of the 152 hands with a grade of 3, 129 (84.9%) had partial thenar atrophy and 23 (15.1%) had total thenar atrophy (Table 8). Of the 52 hands with a grade of 4, cases were evenly divided between total and partial thenar atrophy.

**Figure 7:**
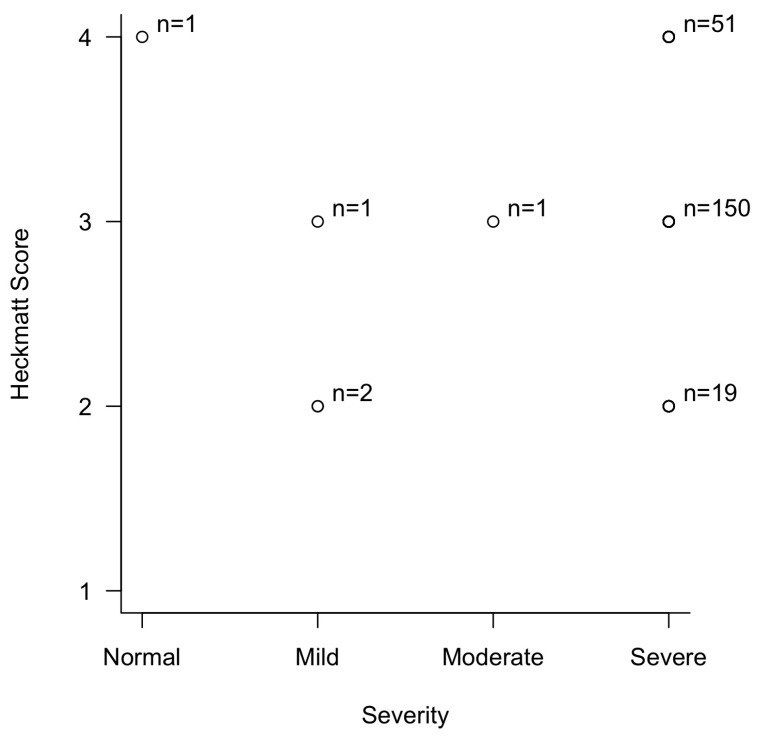
Correlation between the severity of carpal tunnel syndrome and Heckmatt score in patients with thenar muscle atrophy.

The Spearman correlation test using the severity of CTS and Heckmatt score showed an observed Spearman’s *ρ* (rho) of 0.08 (*p* = 0.217) (Figure 7). This statistically insignificant finding was most likely due to the overwhelming majority of patients having severe CTS.

## 4. Discussion

When a patient presents with thenar muscle atrophy, the primary goal is to determine the cause and then formulate the appropriate treatment. The clinical examination focuses on the observation of flattening of the thenar eminence. It also provides anatomical localization of the lesion site by determining whether other muscles such as the first dorsal interosseous (FDI) (innervated by the ulnar nerve) and extensor digitorum communis (EDC) (innervated by the radial nerve) are also atrophic or weak, which would indicate an abnormality in the lower trunk of the brachial plexus, more proximally at the C8 or T1 nerve roots, or ventral horn cells. If the FPL and pronator teres muscles are weak, the location of the lesion is in the proximal median nerve. It is important to assess whether the sensory loss is in the median nerve distribution or in a dermatomal distribution. The next step is to uncover the cause of the thenar muscle atrophy, which may be due to distal median nerve neuropathy such as CTS or median nerve neuropathy at a more proximal location [29], disuse atrophy from CMC joint arthritis, or from congenital thenar aplasia.

In total thenar muscle atrophy, all three muscles (APB, OP, and FPB) are atrophic, as confirmed by weakness documented by clinical examination and typical findings in US. In partial thenar muscle atrophy, atrophy is limited to one or two muscles, confirmed by selective weakness on examination and US findings. While total thenar atrophy may occur in CTS since both heads of the FPB muscle may be occasionally innervated by only the median nerve, it is more common in combined CTS and ulnar nerve neuropathy, brachial plexopathy, C8 or T1 radiculopathies, or a ventral horn disorder. In these cases, some weakness of the FDI, EDC, and FDP muscles may also be observed if carefully looked for. Partial thenar atrophy is frequently seen in CTS with atrophy of the APB and OP muscles, whereas the FPB may not atrophy since it has dual innervation from both the median and ulnar nerves. Partial thenar atrophy may also be accompanied by hypertrophy of the FPB muscle, presumably due to its overuse to compensate for the loss of the OP muscle. In our study, the thenar muscle atrophy of all hands except two was due to median nerve entrapment at the carpal tunnel. Being cognizant that thenar atrophy may arise from causes other than CTS is important, as reflected by the two patients in our study who had disuse atrophy resulting from rheumatoid arthritis in one and cervical radiculopathy of the C8 and T1 nerve roots in the other patient. In these cases, EDX studies were crucial: normal findings in disuse atrophy and denervation in a radicular distribution in cervical radiculopathy.

In cases of thenar atrophy, the EDX studies may be inconclusive due to the total loss of CMAP and SNAP of the median nerve arising from severe entrapment at the carpal tunnel. In this situation, testing with the recording electrode over the second lumbrical muscle may improve the diagnostic yield. However, the CMAP over the second lumbrical muscle may also be absent in more severe cases. Topography of the needle EMG (showing denervation limited to APB and OP muscles) may be helpful, but the study must be extensive by recording from several muscles to rule out proximal median, lower trunk of brachial plexus, and T1 radiculopathies/neuronopathies).

When thenar atrophy is partial, it is possible to determine innervation of the intact portion of the thenar muscles through additional EDX studies. The recording electrode is placed over the thenar area, and the CMAP amplitude following median and ulnar nerve stimulation at the wrist is measured. In cases of severe CTS, there will be no CMAP on median nerve stimulation and normal CMAP on ulnar stimulation. Care should be taken to avoid contamination by CMAP volume conduction from the adjacent adductor pollicis muscle by placing the recording electrode on the radial aspect of the thenar eminence. This test can provide insight into the relative contribution of the median and ulnar nerves in the innervation of the thenar muscles.

US is invaluable in localizing the causative lesion of thenar muscle atrophy, especially when EDX studies are non-localizing due to the absence of CMAPs and SNAPs of the median nerve. A few previous studies have evaluated the benefits of utilizing US in patients with thenar atrophy and CTS [30,31,32]. In Rayegani and colleagues’ study of US and EDX findings in relation to the severity of CTS in 50 patients (96 wrists), there was a statistically significant relationship between the US findings (mean CSA-inlet and WFR) and severity of CTS based on EDX studies [32]. As the severity of CTS increased, the US parameters also increased significantly. In Gronfors and colleagues’ study of 97 hands with CTS that underwent nerve conduction studies (including 53 APB muscles with needle EMG), shear wave velocity correlated positively with the severity of CTS [30]. Mean shear wave velocity was faster in the APB muscles with neurogenic abnormalities compared to those with normal findings. Iida and colleagues also evaluated the elasticity characteristics of thenar muscles in CTS [31]. In their study of 22 patients with CTS compared to controls who underwent US shear wave elastography, the elastic modulus of the APB muscle in the patient group was statistically lower than that in the control group [31].

Initially developed by Heckmatt and colleagues in 1982 [33] with subsequent modifications [25,26], the Heckmatt scoring scale compares muscle echogenicity to nearby bone reflection by US. The scale was originally used to sonographically assess quadricep muscles in pediatric patients with Duchenne muscular dystrophy [33]. The Heckmatt scale can be helpful in the sonographic evaluation of myopathies and various other neuromuscular pathologies, including thenar atrophy [26,34,35]. A higher Heckmatt score is associated with an increase in fibrous tissue in muscle, which provides an indication of the severity of muscle degeneration. In our study, the most frequent Heckmatt grade was 3 in 152 (67.3%) hands, with 204 (90.3%) hands having a grade of 3 or 4. These findings indicate that the majority of patients in our series had either moderately severe or severe thenar muscle degeneration. An additional observation is that the vast majority of patients in this study had severe CTS, which may be due to elderly patients delaying seeking medical attention until their symptoms are severe. Of the 152 hands with a EDX grade of 3, 129 (84.9%) had partial thenar atrophy and 23 (15.1%) had total thenar atrophy. Of the 52 hands with a EDX grade of 4, total and partial thenar atrophy were equal in frequency.

We speculated that partial thenar atrophy (only the radial half of thenar eminence showing atrophy) was due to sparing of the FPB muscle, which is partially innervated by the ulnar nerve. We surmised that if ulnar neuropathy coexists with severe CTS, the FPB muscle will also be atrophic and will lead to total rather than partial atrophy. The FPB muscle may hypertrophy as it presumably compensates for the loss of the OP muscle. The FPB muscle may also be prominent if it is innervated exclusively by the ulnar nerve and may protrude medial to the atrophic APB muscle. On the contrary, the median nerve may innervate both heads of the FPB muscle and lead to total atrophy in severe entrapment at the carpal tunnel. This may also occur if the patient has thenar atrophy from severe CTS in the context of the additional presence of ulnar neuropathy. However, this theory was not confirmed in this study. This may be because all cases of ulnar neuropathy may not have been severe enough to cause significant axon loss, with resultant atrophy of the FPB muscle. A total of 94 (41.6%) hands in our study had co-existing thenar muscle atrophy and ulnar nerve neuropathy, with the majority (59 [62.8%]) having partial thenar atrophy. Fifteen patients had evidence of a triad of partial thenar atrophy, ulnar neuropathy, and FPB muscle hypertrophy. It is possible that the severity of ulnar neuropathy may be an important factor as we excluded cases with weakness and atrophy of ulnar innervated muscles in this study.

### Strengths and Limitations

The strength of the present study is that it involves the largest number of patients with thenar atrophy and highlights the role of both EDX and US studies in arriving at a conclusive diagnosis. Limitations of this study include its retrospective nature and lack of follow-up, as most patients were only evaluated once at our neurodiagnostic center. We were unable to determine whether patients with thenar muscle atrophy underwent a CTR and had improvement of the thenar atrophy postoperatively. Another limitation is that the same physician performed and interpreted the EDX and US findings. Evaluation of the US findings by a blinded/independent specialist was desirable and will be incorporated in future studies.

## 5. Conclusions

In this study of a large cohort of patients with thenar muscle atrophy, we sought to determine the underlying cause using both EDX and US studies. The EDX study was often non-localizing in severe median nerve entrapment at the carpal tunnel. In this situation, US was very useful, confirming severe CTS as the most common underlying cause of thenar muscle atrophy. We also speculated on the mechanism of varied clinical patterns of thenar muscle atrophy: partial, total, and partial with FPB muscle hypertrophy.

## Figures and Tables

**Table 1:** Pattern of thenar atrophy.

Type of Thenar Atrophy	Description	Mechanism
Total	APB, OP, and FPB muscles are atrophic, leading to a “flat” thenar eminence	1. Severe CTS: median nerve innervates all thenar muscles, including both heads of the FPB muscle
		2. Severe CTS and ulnar neuropathy causing atrophy of all thenar muscles, including both heads of the FPB muscle
		3. T1 radiculopathy causing atrophy of all thenar muscles, including both heads of the FPB muscle
Partial	APB and OP muscles are atrophic with an intact FPB muscle visible in the distal/medial part of the thenar eminence	1. Severe CTS with intact deep head of the FPB (innervated by the ulnar nerve)
		2. Severe CTS with an intact FPB muscle due to ulnar innervation of both heads
Partial with hypertrophy of the FPB	APB and OP muscles are atrophic, causing the lateral part of thenar eminence to be flat with a prominent “bulge” on the medial aspect from a large FPB muscle	Severe CTS with an intact FPB muscle compensating for the loss of the OP muscle; more likely when the FPB muscle is entirely supplied by the ulnar nerve

APB: abductor pollicis brevis muscle; OP: opponens pollicis muscle; FPB: flexor pollicis brevis muscle; CTS: carpal tunnel syndrome.

**Table 2:** Clinical findings of patients with thenar atrophy.

Characteristics	Number of Patients (n = 197)
Age (mean)	68.8 years (Range: 15–92 years)
Sex (%)	
Male	97 (49.2)
Female	100 (50.8)
Side (%)	
Right	109 (55.3)
Left	59 (30.0)
Both	29 (14.7)
Dominant hand (%)	
Right	172 (87.3)
Left	22 (11.2)
Ambidextrous	3 (1.5)
Hand dominance corresponds with side of symptoms (%)	
Yes	135 (68.5)
No	62 (31.5)
Diabetes mellitus (%)	
Yes	33 (16.8)
No	164 (83.2)
History of carpal tunnel release = Yes (%)	14 (6.2)

**Table 3:** Neurological examination of patients with thenar atrophy (total number of hands).

	Number of Hands (n = 226)
Thenar atrophy (%)	
Total	52 (23.0)
Partial	174 (77.0)
Prominent FPB = yes (%)	44 (19.5)
Total atrophy	0 (0%)
Partial atrophy	44 (100%)
Weakness (%)	
APB	226 (100.0)
OP	226 (100.0)
FDI	15 (6.6)
ADM	6 (2.6)
FDP	3 (1.3)
EDC	2 (0.88)
FPL	1 (0.44)
EPL	1 (0.44)
Sensation loss = yes (%) *	217 (96.0)

* Sensation: normal in 1 patient and unreliable in 8 patients; FPB: flexor pollicis brevis muscle; APB: abductor pollicis brevis muscle; OP: opponens pollicis muscle; FPL: flexor pollicis longus muscle; FDI: first dorsal interosseous muscle; ADM: abductor digiti minimi muscle; FDP: flexor digitorum profundus muscle; EDC: extensor digitorum communis muscle; EPL: extensor pollicis longus muscle.

**Table 4:** Severity of median nerve entrapment at the carpal tunnel according to electrodiagnostic findings in patients with thenar atrophy (total number of hands).

Severity *	Criteria	Total Number of Hands (n = 226)
Mild	Only sensory fascicles affected	3 (1.3%)
Moderate	Sensory and motor fascicles affected	0 (0%)
Moderately severe	Sensory and motor fascicles affected with motor unit changes (increase in polyphasic units) in APB	1 (0.44%)
Severe	Loss of SNAP and loss of or decrease in amplitude CMAP of APB < 1 mV, along with needle EMG showing denervation of APB	220 (97.3%)

* Two patients did not have evidence of carpal tunnel syndrome according to electrodiagnostic studies. Thenar atrophy was due to disuse atrophy associated with rheumatoid arthritis in 1 patient and cervical radiculopathy of C8 and T1 nerve roots in 1 patient. APB: abductor pollicis brevis muscle; SNAP: sensory nerve action potential; CMAP: compound muscle action potential.

**Table 5:** Motor electrodiagnostic findings in patients with thenar atrophy (total number of hands).

Metrics	Number of Hands (n = 226)
CMAP of APB (%)	
Absent	186 (82.3)
0.1–2.0 mV	36 (15.9)
2.01–4.0 mV	4 (1.8)
>4.0 mV	0 (0)
CMAP of second lumbrical (%) (n = 188)	
Absent	106 (56.4)
0.1–2.0 mV	82 (43.6)
2.01–4.0 mV	0 (0)
>4.0 mV	0 (0)
Distal motor latency of APB (%)	
Absent	186 (82.3)
0.1–6.0 ms	10 (4.4)
>6.1 ms	30 (13.3)
Distal motor latency of second lumbrical (%) (n = 188)	
Absent	106 (56.4)
0.1–6.0 ms	17 (9.0)
>6.1 ms	65 (34.6)
Motor unit recruitment of APB (%) *	
Normal	2 (0.88)
Decreased	104 (46.2)
Absent	119 (52.9)
Fibrillations/positive waves of APB (%) *	
No	53 (23.6)
Yes	172 (76.4)
Ulnar nerve neuropathy on side of thenar atrophy (%) (n = 94 [41.6])	
Total atrophy	35 (37.2)
Partial atrophy	59 (62.8)
Ulnar nerve neuropathy on side of thenar atrophy with partial atrophy (%) (n = 59)	
Without FPB muscle hypertrophy	44 (74.6)
With FPB muscle hypertrophy	15 (25.4)

* Not performed in 1 patient; CMAP: compound muscle action potentials; APB: abductor pollicis brevis.

**Table 6:** Sensory electrodiagnostic findings in patients with thenar atrophy (total number of hands).

	Number of Hands (n = 226)
SNAP amplitude median nerve (%)	
Absent	212 (93.8)
1–10.0 μV	5 (2.2)
10.01–20.0 μV	3 (1.3)
>20.0 μV	6 (2.6)
SNAP latency median nerve (%)	
Absent	212 (93.8)
3.5–6.0 ms	14 (6.2)
>6.0 ms	0 (0)

SNAP: sensory nerve action potential.

**Table 7:** Ultrasound findings in patients with thenar atrophy (total number of hands).

	Number of Hands (n = 226)
APB hyperechoic	
Yes	225 (99.6)
No	1 (0.44)
OP hyperechoic	
Yes	225 (99.6)
No	1 (0.44)
FPB hyperechoic	
Yes	29 (12.8)
No	197 (87.2)
Wrist CSA	
<10 mm^2^	3 (1.3)
10–20 mm^2^	104 (46.0)
>20 mm^2^	119 (52.7)
Forearm CSA	
3–6 mm^2^	102 (45.1)
7–10 mm^2^	104 (46.0)
>10 mm^2^	20 (8.9)

APB: abductor pollicis brevis muscle; OP: opponens pollicis muscle; FPB: flexor pollicis brevis muscle; CSA: cross-sectional area.

**Table 8:** Heckmatt scale determined by ultrasound in patients with thenar atrophy (total number of hands).

Grade	Description	Total Number of Hands (n = 226)	Total Thenar Atrophy (n = 174)	Partial Thenar Atrophy (n = 52)
1	Normal	1 (0.44%)	1 (100%)	0 (0%)
2	Increased muscle echogenicity (with normal architecture) with normal bone reflection	21 (9.3%)	2 (9.5%)	19 (90.5%)
3	Further increased muscle echogenicity (with some loss of architecture) with reduced bone reflection	152 (67.3%)	23 (15.1%)	129 (84.9%)
4	Markedly increased muscle echogenicity (with total loss of architecture) with absent bone reflection	52 (23.0%)	26 (50.0%)	26 (50.0%)

## Data Availability

All of the data for this study is included in the current article.
